# The Non-Coding Regulatory RNA Revolution in Archaea

**DOI:** 10.3390/genes9030141

**Published:** 2018-03-05

**Authors:** Diego Rivera Gelsinger, Jocelyne DiRuggiero

**Affiliations:** Department of Biology, The Johns Hopkins University, Baltimore, MD 21218, USA; dgelsin1@jhu.edu

**Keywords:** small RNAs, sRNAs, non-coding RNAs, ncRNAs, archaea, stress response, gene regulation, RNA-seq

## Abstract

Small non-coding RNAs (sRNAs) are ubiquitously found in the three domains of life playing large-scale roles in gene regulation, transposable element silencing and defense against foreign elements. While a substantial body of experimental work has been done to uncover function of sRNAs in Bacteria and Eukarya, the functional roles of sRNAs in Archaea are still poorly understood. Recently, high throughput studies using RNA-sequencing revealed that sRNAs are broadly expressed in the Archaea, comprising thousands of transcripts within the transcriptome during non-challenged and stressed conditions. Antisense sRNAs, which overlap a portion of a gene on the opposite strand (*cis*-acting), are the most abundantly expressed non-coding RNAs and they can be classified based on their binding patterns to mRNAs (3′ untranslated region (UTR), 5′ UTR, CDS-binding). These antisense sRNAs target many genes and pathways, suggesting extensive roles in gene regulation. Intergenic sRNAs are less abundantly expressed and their targets are difficult to find because of a lack of complete overlap between sRNAs and target mRNAs (*trans*-acting). While many sRNAs have been validated experimentally, a regulatory role has only been reported for very few of them. Further work is needed to elucidate sRNA-RNA binding mechanisms, the molecular determinants of sRNA-mediated regulation, whether protein components are involved and how sRNAs integrate with complex regulatory networks.

## 1. Introduction

Small RNAs (sRNAs) are important regulators for multiple cellular functions and they are ubiquitous in all domains of life. sRNAs, also called non-coding RNAs (ncRNAs), are RNAs that do not function as mRNAs, ribosomal RNAs, or transfer RNAs in the cell. sRNAs in bacteria and eukarya play essential roles in transcriptional regulation, chromosome replication, RNA processing and modification, mRNA stability and translation and even protein degradation and translocation [1,2,3]. Recently, it was discovered that archaeal genomes encode for large numbers of sRNAs and that many of them are responsive to environmental stresses [4,5,6,7,8,9,10,11,12,13,14]. 

While much remains to be elucidated about sRNAs in Archaea, decades of research in Eukarya and Bacteria have built a body of knowledge on their functional roles and their mechanisms of action. In *Eukarya,* several types of sRNAs have been identified, including microRNAs (miRNAs), PIWI-associated RNAs (piRNAs) and endogenous small interfering RNAs (siRNAs) [1]. The most studied of these, miRNAs, regulate protein expression in key cellular processes. miRNAs are typically 20–30 nt long, target the 3′ end of their cognate mRNAs and form complexes with Argonaute (Ago) proteins [15]. While Ago homologs have also been found in archaeal genomes, there is no evidence for eukaryotic-like RNA interference in these organisms [16]. Rather, a defensive role against foreign genetic material was recently proposed whereby archaeal Ago proteins direct guide-dependent cleavage of foreign DNA [17].

**Figure 1:**
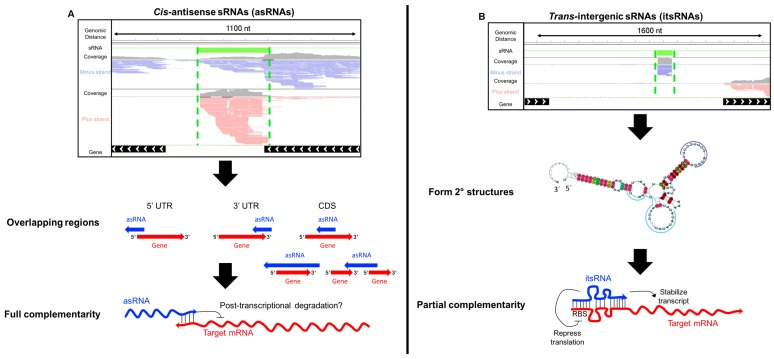
Classes of small non-coding RNAs (sRNAs) discovered in Archaea. Genome viewer of (**A**) antisense sRNAs (*cis*-acting) and (**B**) intergenic sRNAs (*trans*-acting). Paired-end reads (100 bases) were mapped to the *Haloferax. volcanii* National Center for Biotechnology Information (NCBI) reference genome. Reference genes are marked as black lines with white arrows indicating their location on the plus strand (>) or minus strand (<). Reads marked in red are transcribed from the minus strand while blue reads are transcribed from the plus strand. Green lines indicated sRNAs. Coverage plots are in gray. Diagrams of classes of antisense RNAs (asRNAs) are shown based on binding attributes: overlapping the 3’ untranslated region (UTR), the 5′ UTR, within the coding sequence (CDS), extending past the CDS and overlapping multiple mRNAs. An example of an intergenic sRNA secondary structure is shown in (**B**) [5]. Reported regulatory roles of archaeal sRNAs are shown at the bottom.

In bacteria, sRNAs are typically 50 to 300 nt in length and act by targeting mRNA stability, translation, or by binding to proteins [18]. Base-pairing with their mRNAs targets are of two types. *Cis*-encoded antisense RNAs (asRNAs) are encoded on the DNA strand opposite their target gene and thus can act via extensive base pairing (Figure 1A). asRNAs have been found to repress transposons and toxic protein synthesis and to modulate the expression of transcriptional regulators [2,18]. In contrast, *trans*-encoded sRNAs are encoded at genomic location distinct from their target mRNAs, such as intergenic regions and act via limited base pairing [19] (Figure 1B). These sRNAs bind at the 5′ end or 3′ end of their target, either blocking ribosome binding and/or triggering degradation of target mRNAs via the endoribonuclease RNaseE [18]. sRNAs can also activate translation by preventing the formation of inhibitory secondary structures and therefore increasing ribosome binding [2]. *Trans*-encoded sRNAs can target multiple genes, including key transcription factors and regulators and, as a consequence, a single sRNA can modulate the expression of very large regulons [15,18]. A typical example of that is the sRNA *OxyS* involved in the oxidative stress response in *Escherichia coli* [20]. In most Gram-negative bacteria, the RNA binding protein Hfq is required for function and/or stability of the sRNAs [2]. Hfq is structurally related to the Sm/Lsm family of proteins and acts by stabilizing RNAs or by promoting rapid mRNA-sRNA base-pairing and recruiting of RNAseE for degradation [2]. However, other bacteria do not require Hfq for sRNA-mediated regulation even when the protein is encoded in their genome. Recently, novel RNA-binding proteins such as CsrA and ProQ have also been proposed to function as sRNAs chaperones in bacteria [21]. 

In Archaea, the functional and mechanistic characterization of sRNAs is still in its infancy. The size range of archaeal sRNAs is 50 to 500 nt in length. Both *cis-*asRNAs and *trans*-encoded sRNAs (thereafter called intergenic sRNAs (itsRNAs)) have been reported in a number of archaeal species (Figure 1), as well as *cis* sense sRNAs that are transcribed within the open reading frame (ORF) of genes [4,14,22,23,24,25,26,27]. In addition to these ncRNAs, small nucleolar RNAs (snoRNAs), tRNA-derived fragments and CRISPR RNAs (crRNAs) have been found in Archaea. This review will focus on the *cis*- and *trans*-encoded archaeal sRNAs as it becomes more evident that they play essential roles in gene regulation and because there have been exciting new developments in the last few years (since the last sRNA review) in unraveling the functional roles of these sRNAs. We will first document the sRNAs identified so far in the Archaea and discuss the state-of-the art methods for sRNA detection. We will then describe the molecular mechanisms that have been elucidated for a small number of archaeal sRNAs, give an overview of sRNA-interacting partners and as such provide insights into the biological roles of these sRNAs. Lastly, we will discuss the future prospects for archaeal sRNA investigations.

## 2. Identification of sRNAs: What Has Been Found So Far?

In contrast to the wealth of knowledge on bacterial and eukaryal RNA regulators, our knowledge of sRNAs in Archaea is limited to a handful of studies for hyperthermophiles, methanogens and the haloarchaeon *Haloferax volcanii* [9,11,28]. Both classes of *trans*- and *cis*-encoded regulatory sRNAs have been found in the Archaea. Initial identification of sRNAs relied on bioinformatic (RNomics) prediction using archaeal whole genome sequences. Later, microarray and 454-pyrosequencing technologies provided a mean to validate these predicted sRNAs and further identified novel sRNAs by the hundreds. However, it is the unprecedented discovery of more than 2900 sRNAs in *H. volcanii* by two recent high-throughput sequencing (HTS) studies—which is quite remarkable considering that the genome of this organism encodes for just over 4000 proteins [4,14]—that has permanently altered our view of the archaeal transcriptome. From these studies, it is now clear that a large proportion of RNAs are non-coding, nearly rivaling the number of RNAs encoding for proteins [4,14]. Additionally, non-coding RNAs up to 1000 nt in length were also reported [4], thus increasing the size range of identified archaeal non-coding RNAs. However, because of the deep level of sequencing in HTS studies it is important to use thresholding criteria to distinguish small RNAs from transcriptional noise or non-functional transcripts. Such criteria may include the presence of promoter elements, conservation across taxa, minimum expression levels and differential regulation under specific conditions.

In the model haloarchaeon, *H. volcanii*, as many as 1500, asRNAs and 400 intergenic sRNAs have been identified, indicating that most sRNAs in this organism are antisense to coding regions. Furthermore, as much as 30% of the sRNAs discovered in *H. volcanii* contained stringent basal transcriptional promoters, such as a TATA-box and exhibited expression levels comparable to mRNAs, underling their relevance in the global regulation of gene networks [4,14].

While sRNAs are particularly numerous in haloarchaea genomes [14], they have also been found in a number of other Archaea, including *Sulfolobus* [22], *Methanosarcina* [23], *Pyrobaculum* [24], *Pyrococcus* [25], *Thermococcus* [26] and *Methanolobus* [27] (Table 1). In *Sulfolobus solfataricus*, 125 *trans*-encoded sRNAs and 185 *cis*-encoded asRNAs were identified using HTS, suggesting that 6.1% of all genes in *S. solfataricus* are associated with sRNAs [22]. A comparative genome analysis of *Methanosarcina mazei*, *Methanosarcina bakeri* and *Methanosarcina acetivorans* revealed that 30% of the antisense and 21% of the intergenic sRNAs identified were conserved across the 3 species [23]. Similar to bacteria, the number of antisense sRNAs reported in the Archaea numbers in the hundreds and further work is greatly needed to validate and characterize their functional roles [19]. It should be noted that of some the differences in numbers of sRNAs in Table 1 may be attributed to differences in sequencing technologies and sequencing depths used in these studies. In particular, studies that used microarray versus 454-sequencing and HTS with Illumina platforms (Illumina, San Diego, CA, USA), the latter two allowing for de novo discovery of the entire transcriptome. The sRNA numbers reported in Gelsinger et al. [4], Babski et al. [14], Li et al. [27], Jäger et al. [26], and Toffano-Nioche et al. [25] all used HTS Illumina technologies and are thus most comparable with each other. All other studies used microarray or 454-sequencing technologies.

## 3. Best Methods for sRNA Discovery

Methods at the forefront of sRNA discovery in Archaea are all RNA sequencing methods that take advantage of the sequencing depth and high throughput of Illumina technologies. These methods are (1) differential RNA-sequencing (dRNA-seq) and (2) size-selected, strand-specific sRNA-sequencing (sRNA-seq) [14,31].

Differential RNA-seq was used to identify hundreds to thousands of sRNAs in *H. volcanii*, *Methanolobus psychrophilus*, *Thermococcus kodakerensis* and *Pyrococcus abyssi* [14,25,26,27]. The dRNA-seq method is based on the selective enrichment of primary transcripts and allows for transcription start site mapping [32]. This provides a global approach to identify all primary RNAs and the exact position at which they are transcribed, under any condition [32]. However, a significant drawback to this method is that it does not provide information on the length of the sRNAs because it is restricted to the 5′-ends of the transcripts; it is also biased against processed sRNAs [14]. Another method for sRNA identification is presented in Gelsinger et al. (2018) [4] and uses a modified sRNA-seq protocol that enabled strand-specific deep sequencing and identification of thousands of sRNAs in *H. volcanii*. In this method, RNA is size-selected and strand-specificity is preserved [4]. By significantly enriching for sRNAs, this method provides better detection of full length of sRNAs and its strand specificity allowed for the clear identification of antisense and intergenic sRNAs and potential targets of these sRNAs. However, the detection of internal sense sRNAs appeared to be difficult because of their masking by mRNA reads.

Besides library preparation and sequencing, another major difficulty in sRNA identification is in the bioinformatic analysis of the RNA-seq data. While, no single pipeline has been published to specifically identify sRNA in Archaea, the computational strategy used in Gelsinger et al. (2018) presents a significant step forward in designing analytical pipeline specific for archaeal sRNA discovery.

## 4. Molecular Regulatory Mechanisms and Targets of Small Non-Coding RNA in Archaea

### 4.1. Antisense sRNAs 

**Figure 2:**
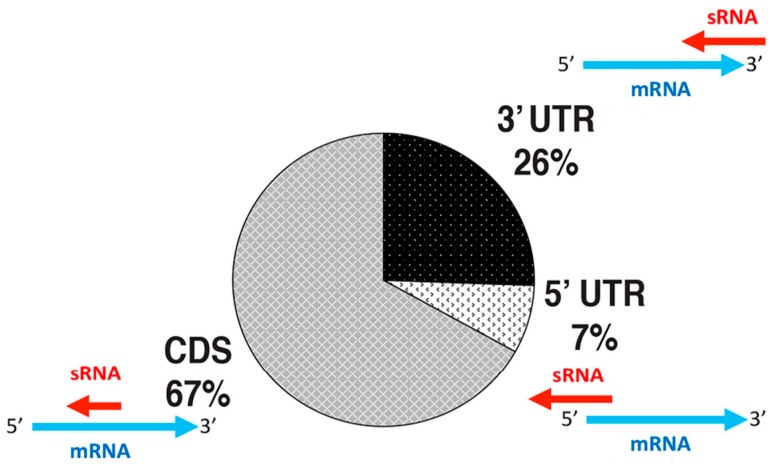
Distribution of binding regions for antisense sRNAs. Colors indicate type of sRNA binding [4]. CDS: coding sequence.

Despite the discovery of thousands of sRNAs in archaeal transcriptomes, functional and mechanistic characterizations of sRNAs in the Archaea is in its infancy. Initial insight into antisense sRNA mechanisms comes from recent work in *H. volcanii,* showing that an overwhelming majority of all sRNAs expressed in this organism are antisense [4,14] (Figure 2). Of these, only a minority (7%) overlapped the 5’ UTR of mRNAs, which is in concurrence with findings that most mRNAs in *H. volcanii* are leaderless (lacking a 5′ UTR) [4], while most (67%) overlapped within the coding sequence (CDS) of mRNAs (Figure 2). In bacterial itsRNAs and eukaryal sRNAs, the region of interaction (hybridization) between a sRNA and its target mRNA has been termed a *seed* region [4]. In *H. volcanii* no *seed* binding region for CDS-binding sRNAs have been found, indicating that they could have full occupancy upon the mRNA [4]. However, while thermodynamically favorable, the hybridization across the full length of CDS-asRNAs has not been demonstrated in vivo. Additionally, some of these CDS-asRNAs have the potential to form a secondary structure, suggesting that the CDS-asRNA might only bind part of the transcript (a partial *seed* region). Lastly, a smaller fraction (26%) of asRNAs overlapped the 3′ UTR of mRNAs [4] (Figure 2).

There are many known advantages of sRNA regulators including reduce metabolic cost, additional levels of regulation, unique regulatory properties and faster response to stresses. Indeed, the regulatory effects of sRNAs are often observed within minutes in bacterial systems [2]. Furthermore, sRNAs in bacteria can regulate very large gene networks as well as key transcription factors [2]. Examples of these include OxyS and SgrS in *E. coli*, involved in oxidative and glucose-phosphate stress, respectively [20]. Antisense sRNAs, which are by far the largest group of sRNAs found in the Archaea, are encoded in the opposite strand of their putative target. In the hyperthermophile *Pyrobaculum*, three antisense sRNAs were found opposite a ferric uptake regulator, a triose-phosphate isomerase and transcription factor B, supporting a potential role in the regulation of iron, transcription and core metabolism [24]. Target enrichment of asRNAs differentially regulated by oxidative stress in *H. volcanii* included mRNAs involved in transposon mobility, chemotaxis signaling, peptidase activity and transcription factors [4]. The functional enrichment of transposon targeted by asRNAs suggests that during oxidative stress transposon activity is tightly regulated in *H. volcanii*, potentially explaining its increased resistance to oxidative stress conditions [4]. Indeed, transposons are genetic elements that hop around in the genome causing double strand breaks. This added stress would likely be detrimental to a cell under oxidative stress, hence a need to be silenced [33,34,35]. sRNAs antisense to transposons were also reported for *Thermococcus kodakarensis* [26], *S. solfataricus* [22] and *M. mazei* [23] suggesting that, similarly to bacteria, regulation of transposition is mediated by asRNAs in Archaea [36]. Initial mechanistic insight of asRNAs comes from a recent study of sRNAs in *H. volcanii* by Gelsinger, et al. [4] which found that a large number of asRNAs were either upregulated or downregulated during oxidative stress, revealing two types of antisense sRNA populations. An anti-correlation was observed for a group of upregulated antisense sRNAs and their downregulated *cis*-encoded putative targets, indicating a potential mechanism of negative regulation [4]. In contrast, many *cis*-encoded putative mRNA targets and their cognate asRNAs exhibited a positive correlation in their expression patterns to oxidative stress, suggesting a positive regulatory effect between asRNA-mRNA *cis*-pairs [4]. Although negative regulatory effect of asRNAs on their target mRNAs was also suggested in another study, also in *H. volcanii* [14], experimental evidence is still lacking because of the inherent difficulty at manipulating such overlapping sRNA-mRNAs pairs. 

### 4.2. Intergenic sRNAs

While the regulatory effects of asRNAs can be readily inferred because of the overlap with their mRNAs targets, it is rather different with intergenic sRNAs where finding targets is a particularly difficult task. As a consequence, mechanistic insights into the regulation of intergenic sRNAs have only been provided for very few specific sRNAs. In *H. volcanii*, many intergenic sRNAs are differentially expressed in response to varying environmental conditions, including elevated temperature, osmotic stress, nutrient limitation and oxidative stress, [4,8,12,37,38]. While phenotypic characterization of sRNA deletion mutants, including 10 gain-of-function phenotypes out of 27 mutants tested, confirmed their roles in metabolic regulation, stress adaptation and complex behavior [12,38], their targets are still unknown with a few exceptions. 

**Figure 3:**
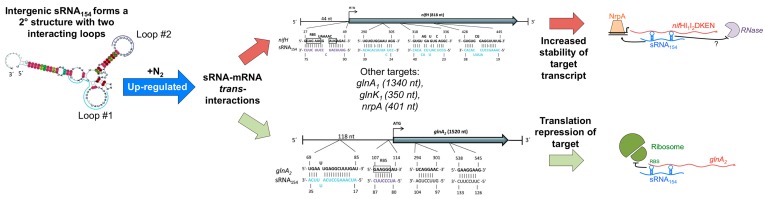
Two proposed mechanisms of action and targets for the intergenic sRNAs (itsRNA), sRNA154, in *M. mazei* under conditions in which N2 is the only source of nitrogen modified from [5]. sRNA154 is predicted to form a stable secondary structure with two stem loops that interact with mRNA targets. Mechanism 1 (**top**): target stabilization by sRNA-mediated masking of sites for degradation by an unknown RNase for the *nif* operon and the transcription factor *nrp*A, which regulate the *nif* operon. Mechanism 2 (**bottom**): sRNA154 binds to the glnA2 ribosome binding site (RBS) via loop #2, preventing translating and thus decreasing the amount of protein produced but not that of the transcript.

Some of these exceptions comes from the study of *M. mazei* cultures grown under nitrogen starvation conditions where RNA-seq experiments revealed the differential expression of a number of sRNAs in response to nitrogen availability [23,39]. This then resulted in the identification of the first in vivo targets for archaeal intergenic sRNAs [23,39]. A potential target for one of these sRNAs, sRNA_162_, was a bicistronic mRNA encoding for a transcription factor involved in regulating the switch between carbon sources and for a protein of unknown function [39]. Another sRNA, sRNA_154_, was also exclusively expressed during nitrogen starvation conditions and the multiple targets for sRNA_154_ included mRNAs for the α subunit of nitrogenase (*nif*H), the transcriptional activator of the *nif* operon (*nrp*A) and glutamine synthase1/2 (*gln*A_1_/*gln*A_2_). Prasse et al. [5] determined that sRNA_154_ stabilized some mRNAs while inhibiting translation initiation for other mRNAs, thus playing a dual regulatory role (Figure 3). sRNA_154_ was found to stabilize nifH-, nrpA- and glnA_1_-mRNAs but to block the translation of *glnA_2_*-mRNA. sRNA_154_ is highly conserved in the *Methanosarcina* and it was predicted to form a stable secondary structure with two loops required for stabilization of mRNA targets. The authors of the study proposed that the mechanism of the two loops was to mask endonucleolytic cleavage sites of RNases by hybridizing to the mRNAs target and, thus, preserving the mRNA for translation [5]. In contrast, they also showed that loop 2 of sRNA_154_ contains anti-ribosome binding site (RBS) sequences that masked the RBS of the *glnA*_2_-mRNA target, repressing translation initiation [5]. Thus, the proposed functional role of sRNA_154_ was to regulate N_2_-fixation under nitrogen limiting conditions by stabilizing transcripts involved in nitrogenase production (both regulators of and the nitrogenase itself), leading to a feed forward regulatory system [5]. 

Most recently, another *M. mazei* itsRNA, sRNA_41_, was found to be downregulated during nitrogen limiting conditions. Targets of sRNA_41_ were involved in acetyl-CoA-decarbonylase/synthase complexes (ACDS) and were repressed at the translational level [13]. Thus, sRNA_41_ was predicted to play a role in repressing ACDS protein levels, however, under nitrogen limiting conditions, sRNA_41_ was downregulated, thus allowing ACDS levels to increase, which in turn provided sufficient amino acids for nitrogenase synthesis and energy for N_2_-fixation [13]. Thus, the proposed mechanism of regulation for sRNA_41_ was to repress its targets at the translation level by masking ribosome binding sites within polycistronic mRNAs [13]. Other molecular mechanisms have been identified in the Archaea such as the binding of itsRNAs to the 3’UTR of mRNA targets in *S. solfataricus* [40]. This is of particular interest in the *Haloarchaea* because 72% of their transcripts are leaderless [14].

**Figure 4:**
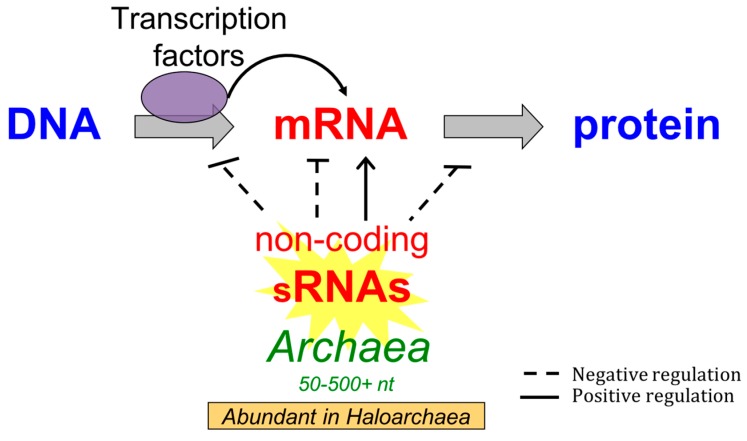
Correlation- and experimental-based regulatory mechanisms for sRNAs reported in the *Archaea*. The grey arrows indication the processes of transcription and translation. Regulatory arrows indicate regulation at transcription, post-transcription and translation steps.

While these studies provide great examples of gene network regulated by sRNAs in the Archaea, additional work is needed to identify many more molecular targets of archaeal itsRNAs and the diverse mechanisms of their sRNA-mRNA interactions. Taken together these studies are building a narrative of sRNA (both antisense and intergenic) mechanisms in the Archaea, combining global approaches with individual targeted sRNA studies, demonstrating that sRNAs are also essential partners in gene regulation in the third domain of life (Figure 4).

## 5. Future Prospects

To date, very few of the newly reported candidate sRNAs in the Archaea have been functionally characterized [5,13,39] and many questions remain: *what are the targets of the multitude of sRNAs discovered in the Archaea? What are the regulatory effects of these sRNAs? And more importantly, what type* *of molecular mechanisms can we expect in a domain of life where information processing systems are a mosaic of bacterial and eukaryal systems* [41]?

Target identification of sRNAs, especially itsRNAs, is a difficult task due to partial base pairing with multiple targets by a single sRNA. High-throughput strategies have recently been developed in bacteria and eukarya to directly identify RNA-RNA duplexes in vivo using MS2 hairpins (MAPS) [42] or psoralen-mediated crosslinking. One of these methods, sequencing of psoralen crosslinked ligated and selected hybrid (SPLASH) [43] involves five essential steps: (1) specific in vivo cross-linking of RNA-RNA duplexes using psoralen (or derivatives); (2) enrichment of duplexes using biotin-streptavidin methods and degradation of non-duplexed RNA; (3) ligation of an adapter loop to form a chimeric RNA molecule; (4) reverse crosslinking; and (5) high-throughput sequencing of the chimeric RNAs. In contrast to alternative methods for RNA-RNA interactions, such as crosslinking, ligation, and sequencing of hybrids (CLASH) and crosslinking immunoprecipitation (CLIP) [44], SPLASH does not require an sRNA-interacting protein in complex with the RNA duplexes, a component that is still unresolved in the Archaea. Research from these approaches can potentially identify entire sRNA regulon and provide information on sRNA-mRNA *seed* regions, landscape and structural binding motifs that can be used to build archaeal-specific sRNA target prediction models. Potential problems to these methods are that psoralen preferential cross-linking might result in missing targets, false positive, and/or masking of lowly expressed sRNAs by highly abundant RNAs and their interactions. Despite these drawbacks, these methods are a step forward and they provide useful tools for sRNA biology.

Co-immuno-precipitation with the Lsm protein, the archaeal homolog of Hfq, was used to *capture* sRNAs in vitro [6]. However, the role of Lsm, or any other RNA-binding protein-remains to be elucidated in the Archaea. Homologs of eukaryotic miRNA interacting proteins (Ago) have been found in the Archaea but rather than RNA interference, a defensive role against foreign genetic elements has been proposed [45]. Ribonuclease degradation of mRNAs mediated by sRNAs is a hallmark of bacterial sRNA-mRNA regulation. Ribonucleases have been found to play large-scale roles in 5′-3′ directed mRNA decay in the Archaea, including enzymes such as archaeal cleavage and polyadenylation specificity factor (aCPSF2) in *Sulfolobus acidocaldarius* and RNase J in *Methanococcus jannaschii* [46], which raises the question of whether there is an intersection between these RNases and sRNA regulation. Some Archaea have very short or no 5′ UTRs on mRNAs, such as *H. volcanii*, suggesting that the 5′ UTR in these Archaea do not carry information regarding translation initiation or transcript degradation. Indeed, only a small portion (7%) of asRNAs in *H. volcanii* overlap with the 5′ UTR of targets and, therefore, other regions such as the 3′ UTR, may carry the information needed for sRNA-mediated regulation [4]. The observation that the majority of asRNAs in *H. volcanii* overlap within the CDS of targets [4] could indicate that rather than an RNase with exoribonucleolytic activity (aCPSF2/aRNaseJ) interacting with sRNA-mRNA duplexes, an RNase with endoribonucleolytic activity, such as CPSF1 in *M. jannaschii*, could be the major interacting RNase [46]. In this model, the endo-RNase can cleave mRNAs within the CDS, providing a way for either target stabilization, with the sRNA-bound target mRNAs masked from endo-RNase activity, or target degradation, with the sRNA-mRNA duplexes acting as a signal for endo-RNase degradation. Despite the presence of RNases in Archaea, the processing pathways of such enzymes remain to be elucidated and there is no evidence that these proteins interact and/or bind sRNAs in Archaea. Therefore, questions about what ribonucleoprotein complexes are involved in archaeal sRNA regulation and their mechanistic roles remain unanswered. 

Outstanding questions also remain regarding the role of more than 1100 *cis*-sense sRNAs recently discovered in *H. volcanii* [14], the prevalence of regulatory tRNA-derived fragments in the Archaea [8,47] and the potential for sRNAs to encode small peptides such as in *Bacteria* and *Eucarya* [48]. Finally, in vivo quantitative measurements of sRNA-mediated regulation, such as those currently being made in the *Bacteria* [49], are necessary to understand, at a system-level, how sRNA-based regulation is integrated within a cell’s regulatory networks.

## Figures and Tables

**Table 1:** Summary of small non-coding RNAs (sRNA or ncRNA) discovered in the Archaea.

	Number of Genes	Total # sRNAs	# itsRNAs	# asRNAs	# iRNAs	Reference
***Euryarchaeota***						
*Haloferax volcanii*	4023	1557	77	1480	N/A	[4]
*Haloferax volcanii*	4023	2792	395	1244	1153	[14]
*Haloferax volcanii*	4023	190	145	45	N/A	[8]
*Methanolobus psychrophilus*	2974	2745	195	1110	1440	[27]
*Methanosarcina mazei*	3551	242	199	43	N/A	[23]
*Thermococcus kodakarensis*	2328	1731	69	1018	644	[26]
*Pyrococcus abyssi*	1969	322	107	215	N/A	[25]
*Archaeoglobus fulgidus*	248	45	9	33	3	[29]
***Crenarchaeaota***						
*Sulfolobus solfataricus*	3254	310	125	185	N/A	[22]
*Pyrobaculum aerophilum, arsenaticum, calidifontis, & islandicum*	2706, 2407, 2200, 2075	# Not Reported	# Not Reported	3	N/A	[24]
***Nanoarchaeaota***						
*Nanoarchaeum equitans*	553	# Not Reported	# Not Reported	# Not Reported	N/A	[30]

asRNAs: antisense sRNAs; itsRNAs: intergenic sRNAs; iRNAs: internally transcribed sRNAs; N/A: these types of sRNAs were not reported.
